# Vehicle Trajectory Prediction Method for Task Offloading in Vehicular Edge Computing

**DOI:** 10.3390/s23187954

**Published:** 2023-09-18

**Authors:** Ruibin Yan, Yijun Gu, Zeyu Zhang, Shouzhong Jiao

**Affiliations:** College of Information and Cyber Security, People’s Public Security University of China, Beijing 102600, China; yanruibin@stu.ppsuc.edu.cn (R.Y.); 201921420027@stu.ppsuc.edu.cn (Z.Z.); 17605346552@163.com (S.J.)

**Keywords:** edge computing, trajectory prediction, task offloading, vehicle trajectory

## Abstract

Real-time computation tasks in vehicular edge computing (VEC) provide convenience for vehicle users. However, the efficiency of task offloading seriously affects the quality of service (QoS). The predictive-mode task offloading is limited by computation resources, storage resources and the timeliness of vehicle trajectory data. Meanwhile, machine learning is difficult to deploy on edge servers. In this paper, we propose a vehicle trajectory prediction method based on the vehicle frequent pattern for task offloading in VEC. First, in the initialization stage, a T-pattern prediction tree (TPPT) is constructed based on the historical vehicle trajectory data. Secondly, when predicting the vehicle trajectory, the vehicle frequent itemset with the largest vehicle trajectory support is found in the vehicle frequent itemset of the TPPT. Finally, in the update stage, the TPPT is updated in real time with the predicted vehicle trajectory results. Meanwhile, based on the proposed prediction method, the strategies of task offloading and optimization algorithm are designed to minimize energy consumption with time constraints. The experiments are carried out on real-vehicle datasets and the Capital Bikeshare datasets. The results show that compared with the baseline T-pattern method, the accuracy of the prediction method is improved by more than 10% and the prediction efficiency is improved by more than 6.5 times. The vehicle trajectory prediction method based on the vehicle frequent pattern has high accuracy and prediction efficiency, which can solve the problem of vehicle trajectory prediction for task offloading.

## 1. Introduction

In recent years, edge computing-related research has gradually received attention from researchers and scholars [1,2]. Vehicular edge computing (VEC), as a part of edge computing, provides users with real-time service, and has excellent prospects in the fields of intelligent transportation systems, smart city applications, and in-vehicle applications. Meanwhile, how to provide users with the higher quality of service (QoS) of VEC becomes one of the challenges. The QoS still cannot be improved significantly, and one of its bottlenecks is the inefficient task offloading. Traditional task offloading methods which offload the tasks from the vehicles to the cloud server have considerable communication delay [3,4]. Compared with these methods, a lot of research improved task offloading methods through vehicle-to-vehicle (V2V) and vehicle-to-infrastructure (V2I) communications, which reduce the distance of the task transmission [1]. Moreover, resource allocation [5] and network slicing [6] are optimized for task offloading in VEC.

Meanwhile, a variety of task offloading schemes based on trajectory prediction have been proposed to optimize task offloading. For example, high-computation-complexity tasks are computed on the nearby edge servers or the cloud server, and the results are transmitted to another edge server in the direction of the vehicle’s movement through multihop transmission. Once the vehicle enters the transmission range of this server, the computation results are downloaded by the vehicle [7]. However, existing prediction schemes focus on the energy consumption optimization [8,9,10] and ignore the importance of prediction efficiency and accuracy. However, efficient and accurate prediction results are essential to predictive-mode task offloading in VEC.

There are very limited works on the prediction results for multihop transmission in VEC [11,12]. As a result, we have extended existing research and deeply explored the trajectory prediction algorithms. Recently, some studies have used deep learning methods to predict vehicle trajectories including the recurrent neural network (RNN) method [13] and the Transformer method based on the attention mechanism [14]. These deep learning methods require a large amount of memory space and training time. However, the current performance of edge servers is not capable of deploying high-complexity deep learning algorithms [15,16]. At the same time, deep learning algorithms take a long time to predict vehicle trajectories, which cannot meet the demand for real-time prediction [15]; this is discussed in the experiment part. Refs. [11,12] ignore the time consumption of the training process and the prediction process. Trajectory prediction methods based on a frequent pattern do not require large computation and storage resources [17]. These methods also have high accuracy or efficiency. For example, vehicle trajectories can be predicted in real time by constructing T-pattern tree [18,19,20,21], but they have low accuracy. The frequent pattern prediction method proposed in [22] has a higher accuracy rate, but the method is not efficient enough to predict the vehicle trajectory in real time. Therefore, existing trajectory prediction methods based on frequent patterns are not compatible with accuracy and efficiency. Meanwhile, the existing frequent pattern method has to store all of the historical trajectories which take up a lot of storage space.

In order to solve the vehicle trajectory prediction problem in task offloading, this paper designs a T-pattern prediction tree (TPPT) and proposes a real-time vehicle trajectory prediction framework based on frequent patterns. Based on the prediction framework, task offloading strategies and an optimization algorithm are designed.

The main contributions of this work are summarized as follows:

1. We define a new TPPT data structure for trajectory prediction in VEC. To reduce the storage resources in VEC, the TPPT only stores the trajectories related to the current edge server. To improve the accuracy and efficiency, the TPPT stores the vehicle frequent item and the vehicle frequent pattern. The TPPT is updated in real time according to the feedback of the prediction result transmitted from the predicted edge server.
2. In order to improve accuracy and efficiency when using the TPPT in VEC, we propose a TPPT construction algorithm, a TPPT prediction algorithm and a TPPT updating algorithm, respectively. By analyzing the characteristics of edge computing servers, we apply the TPPT in this scenario with the aim of improving efficiency and accuracy. At the same time, it provides real-time prediction results for task offloading.
3. We design the task offloading strategies via V2I and V2V communication based on the proposed prediction. By analyzing the energy consumption, we propose a search algorithm for task offloading to minimize the energy consumption with the constraint of time consumption.
4. Experiments are carried out on real-vehicle datasets [23] and Capital Bikeshare datasets [24] to verify that the vehicle trajectory prediction based on the vehicle frequent pattern has high accuracy and efficiency, which optimizes task offloading in VEC in real time.

The remaining of this paper is organized as follows. Section 2 introduces the relevant literature, definitions and prediction problem. Section 3 presents the data structure and algorithms of trajectory prediction for task offloading in the VEC scenario. The experimental and performance results are included in Section 4. Section 5 concludes this study and points out our future work.

## 2. Literature Review and Problem Statement

### 2.1. Task Offloading Research

**Figure 1:**
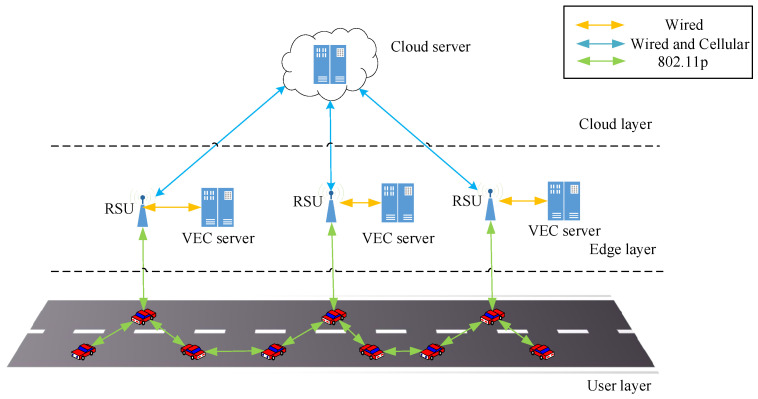
The example of vehicular edge computing.

Task offloading in VEC is a process of transmitting the computation tasks and related parameters from the service requestor to the service provider through V2V or V2I communication. Figure 1 shows different layers of a VEC framework [25].

Optimizing task offloading can make full use of the resources of edge servers and provide a higher QoS to users. One of the most important methods of task offloading is predictive-mode transmission. First, Zhang et al. [7] proposed a predictive offloading framework where the computation tasks were sent to the edge servers ahead of their running direction. The existing improvements took delay and energy consumption into account. For example, Liu et al. [8] minimized the overall energy consumption of task offloading in VEC. The authors also proposed a dynamic programming-based predictive algorithm (DPA) to solve the optimization problem. Furthermore, He and Tian [9] constructed a mathematical optimization model with the constraints of time delay and power consumption for minimizing the power overhead. Combined with V2V and V2I communication, Wang et al. [10] proposed a hybrid multihop edge-computing offloading (VCMO) algorithm. However, there are very limited works on the prediction results which decide the direction of the transmission.

These methods need to predict trajectories in real time. For example, in V2I or V2V, when the tasks or results are transmitted by multihop RSUs [8] or vehicles [10], the trajectory prediction result determines whether the computation result can be downloaded by vehicles in real time. Though deep learning is always used to predict trajectories, Refs. [15,16] pointed out the computation and memory resources of current edge servers could not support the deployment of deep learning for trajectory prediction. At the same time, when the computation tasks are completed and the results are transmitted to the predicted RSU, vehicles have to download the result of computation tasks within the transmission range of the RSU. Therefore, how to predict vehicle trajectories more accurately and efficiently is an urgent problem in the task offloading of VEC.

### 2.2. Trajectory Prediction Research

Trajectory prediction problem can be categorized based on the type of trajectory data into short-distance trajectory prediction, long-distance trajectory prediction and location prediction. For short-distance trajectory prediction, Adam et al. [26] and Huang et al. [27] proposed trajectory prediction methods to avoid collisions for vehicles and humans, respectively. For long-distance trajectory prediction, Wang et al. [28] proposed a multiuser multistep trajectory prediction method which incorporated long short-term memory (LSTM) and sequence-to-sequence (Seq2Seq) learning. For location prediction, Li et al. [29] proposed a prediction framework which integrated individual travel behavior and collective preferences for next location prediction. Different from the traditional trajectory prediction problem, the trajectory prediction of task offloading belongs to the node prediction problem. Namely, the prediction results are the location of the edge servers that the vehicle will pass through in the future.

Recently, research on node prediction has often used deep learning methods to train trajectory data fused with node information. Huang et al. [30] proposed the Bayonet-Corpus which is a trajectory prediction algorithm based on the context of traffic intersections. This algorithm used a Bi-GRU to model the trajectory matrix for the purpose of prediction. Liu et al. [31] proposed a prediction model based on K-nearest neighbors (KNN) to realize real-time urban flow prediction. However, the deep learning approach has a large time complexity and space complexity, which is not suitable for deployment in edge servers. Considering the limited resources of edge servers, trajectory prediction based on frequent patterns is more suitable in task offloading.

Trajectory prediction based on frequent patterns is one of the current research hotspots. First, Monreale et al. [17] proposed WhereNext, a trajectory prediction method based on the T-pattern tree. The tree is a collection of T-patterns which are the frequent patterns of spatiotemporal trajectories. Dong et al. [18] proposed RTMatch, an improved real-time trajectory prediction method based on a real-time pattern tree (RTPT) and a hash table (HT). Comito [21] constructed a frequent-pattern tree, which is similar to a T-pattern tree. Refs. [19,20,22] used frequent trajectory patterns as one part of the trajectory prediction to address the prediction problem. However, current frequent pattern algorithms do not consider the local features of trajectories, which cannot guarantee both accuracy and efficiency.

### 2.3. Definitions and Problem Statement

This section gives the basic definitions of the vehicle trajectory prediction in VEC. Then, the prediction problem statement is given based on these definitions.

**Definition** **1.** *Vehicle Trajectory. A vehicle trajectory is a sequence of a vehicle v passing through n edge servers b according to temporal order, which is mathematically defined as follows*:

(1) $$TB=\;<b_{0},b_{1},\ldots ,b_{k},\ldots ,b_{n}>,n\geq 1$$

*where TB denotes the vehicle trajectory of vehicle v; b_i_ denotes the edge server through which vehicle v passes. The length of the vehicle trajectory TB is n + 1. Simulating the vehicle trajectory with the Capital Bikeshare datasets [24] and assuming that the vehicle trajectory of member number W00742 is TB_0_, we have*

(2) $$TB_{0}=\;<“21\mathrm{st}\;\mathrm{St}”,\ldots ,”14\mathrm{th}\;\mathrm{St}”>$$

**Definition** **2.** *Vehicle Trajectory Support. The vehicle trajectory support s is the frequency of the vehicle subtrajectory TB_i_ in a given vehicle trajectory set D. It is defined as*

(3) $$s_{i}=\mathrm{Support}\left(TB_{i}\right)=|\{TB_{i}|TB_{i}\subseteq TB_{j},TB_{j}\in D\}|$$

*where the vehicle trajectory set D consists of several vehicle trajectories, i.e., D = {TB_1_, TB_2_, …, TB_j_, …, TB_z_}, where the vehicle trajectory TB_j_ is an element in the set D. The length of the vehicle trajectory set D is z. If the vehicle trajectories TB_i_ and TB_j_ satisfy TB_i_ ⊆ TB_j_, TB_i_ is the vehicle subtrajectory of TB_j_*.

Based on the above definitions, this paper gives the definition of the vehicle T-pattern.

**Definition** **3.** *Vehicle T-Pattern. The vehicle T-pattern is a binary tuple for describing the vehicle trajectory and the corresponding vehicle trajectory support, which can be expressed as TP = (TB, E), where TB denotes the vehicle trajectory in Equation (1); E denotes the sequence consisting of the edge R_h_ between neighboring edge servers. Each edge R_h_ consists of neighboring edge servers and the corresponding vehicle trajectory support, i.e., E = <R_1_, R_2_, …, R_h_, …, R_n_>, R_h_ = (b_h−1_, b_h_, s_h_), s_h_ = Support(TB_h_). In the sequence, the length of R is n, when the length of TB is n + 1. Moreover, another representation of the vehicle T-pattern is given in this paper*:

(4) $$TP=\left(TB,E\right)=b_{0}\overset{\mathrm{Support}\left(TB_{1}\right)}{\to }\ldots \overset{\mathrm{Support}\left(TB_{n}\right)}{\to }b_{n}$$

*where TP consists of n + 1 edge servers and n edges. Also taking the Capital Bikeshare datasets as an example, the vehicle T-pattern corresponding to vehicle trajectory TB_0_ is*

(5) $$TP_{0}=\left(TB_{0},E_{0}\right)=“21\mathrm{st}\;\mathrm{St}”\overset{1}{\to }\ldots \overset{1}{\to }”14\mathrm{th}\;\mathrm{St}”$$

According to the definition of the vehicle T-pattern, the prefix of this vehicle T-pattern is defined as the portion of the vehicle T-pattern consisting of the first *n* edge servers, formalized as Front(*TP*). The suffix of the vehicle T-pattern is defined as the (*n* + 1)th edge server, formalized as Back(*TP*). Thus, the prefix and suffix of the vehicle T-pattern in Definition 3 are, respectively,

(6) $$\begin{array}{cc} \mathrm{Front}\left(TP\right) & =b_{0}\overset{\mathrm{Support}\left(TB_{1}\right)}{\to }\ldots \overset{\mathrm{Support}\left(TB_{n-1}\right)}{\to }b_{n-1}\\ \mathrm{Back}\left(TP\right) & =b_{n} \end{array}$$

From the above definition, it is clear that there exists a derivation relation between the prefix and suffix of the vehicle T-pattern, which is also called the vehicle frequent pattern. For given sets *TP*s of vehicle T-patterns, the vehicle frequent pattern is formally defined as

(7) U={ (Front(TP),Back(TP)) | TP in TPs} 

where we also write the elements of *U* as “Front(*TP*) => Back(*TP*). The derivation relation *U* reflects the strength of the derivation relation. Compared with the traditional frequent pattern, no threshold is set on the vehicle frequent pattern.

Finally, based on the existing definitions above, this paper gives the vehicle trajectory prediction problem statement in VEC.

Given:

1. The set *D* = {*TB*_1_, *TB*_2_, …, *TB_j_*, …, *TB_z_*} is extracted from the vehicle trajectory datasets, where any element *TB_j_* is a vehicle trajectory;
2. The sequence *TB_p_* = <*b*_1_, *b*_2_, …, *b_k_*, …, *b_p_*> is the current vehicle trajectory, where any element *b_k_* is an edge server, and its extension *b_p_*_+1_ is to be predicted.

Constraints:

1. The vehicle trajectory *TB_p_*_+1_ consists of the vehicle trajectory *TB_p_* and the unknown but existing edge server *b_p_*_+1_;
2. The vehicle trajectory *TB_p_*_+1_ is a subset of the elements in the vehicle trajectory set *D*. That is, ∃*TB_j_* ϵ *D* such that *TB_p_*_+1_ ⊆ *TB_j_*.

Optimization objective:

Find the edge server node *b_p_*_+1_ such that the vehicle trajectory *TB_p_* and the edge server *b_p_*_+1_ form the vehicle trajectory *TB_p_*_+1_. Meanwhile, the vehicle trajectory *TB_p_*_+1_ has the maximum vehicle trajectory support.

The formal optimization problem is expressed as follows,

(8) $$\underset{b_{p+1}}{\mathrm{argmax}}\;\mathrm{Support}\left({TB}_{p+1}\right)\;s.t.b_{p+1}\in {TB}_{p+1},{TB}_{p+1}\subseteq {TB}_{j},{TB}_{j}\in D$$

## 3. Materials and Methods

In order to accomplish the optimization problem given in this paper, a vehicle trajectory prediction framework based on the vehicle frequent pattern is proposed and this framework is introduced with VEC. This framework is divided into three parts: initializing the edge computing server, predicting the vehicle trajectory, and updating the T-pattern prediction tree in real time. Specifically, the details of the three parts are as follows:

1. We construct the TPPT on the edge server in the initialization stage of the VEC scenario. We propose a TPPT construction algorithm which constructs the vehicle T-pattern tree, vehicle frequent itemset and vehicle frequent pattern based on the historical vehicle trajectories on the edge server locally.
2. We predict the vehicle trajectory based on the TPPT in the VEC scenario. We propose a TPPT prediction algorithm which predicts the future location of the edge server in real time. The computation tasks are transmitted to the prediction result via V2I or V2V communication for the task offloading optimization of VEC.
3. We update the TPPT based on the feedback of prediction results in the VEC scenario. In order to maintain the timeliness of the TPPT in VEC, we propose a TPPT updating algorithm which updates the vehicle frequent itemset and vehicle frequent pattern of the TPPT in real time according to the feedback of the prediction result transmitted from the predicted edge server.

**Figure 2:**
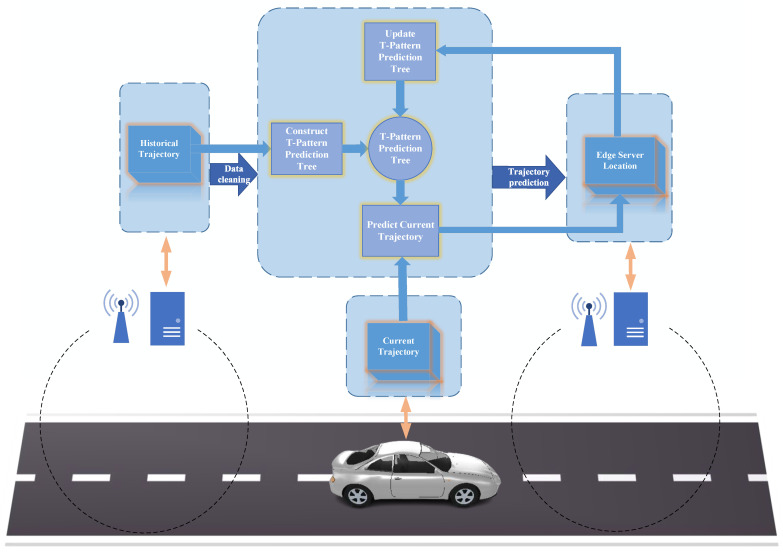
Framework of the vehicle trajectory prediction based on the vehicle frequent pattern.

The vehicle trajectory prediction framework based on the vehicle frequent pattern is shown in Figure 2.

In Figure 2, the arrows do not indicate direct transmission relationships between the vehicle (edge servers) and flow chart. The historical trajectories are stored on edge servers. These trajectories are updated in the updating process. The current trajectory is provided by the vehicle. The edge server location which is the prediction result indicates the name or the serial number of an edge server.

The storage and computation resources are limited in the task offloading of VEC. Meanwhile, predictive-mode task offloading in VEC requires real-time vehicle trajectory prediction. Therefore, in order to ensure the efficiency and accuracy of vehicle trajectory prediction, this paper designs a T-pattern prediction tree (TPPT), a data structure that stores only the historical vehicle trajectories and the vehicle frequent pattern related to the current edge server. The framework shown in Figure 2 mainly solves the optimization problem of this paper through the T-pattern prediction tree.

The TPPT can be deployed in both V2I and V2V communications of VEC. In the initialization stage of V2I communication, the edge server constructs the TPPT based on historical vehicle trajectories. Then, the current trajectory and computation tasks in a vehicle are offloaded to the edge server when the vehicle enters the transmission range of the edge server. Based on the TPPT, the edge server predicts the location of another edge server that the vehicle will pass through in the future. After that, the edge server processes the computation tasks or transmits these tasks to other edge servers or cloud server according to the complexity of the tasks and load balance. If the tasks are completed, the results are transmitted to the predicted edge server. Once the vehicle arrives within the transmission range, the results are downloaded by the vehicle. Finally, the edge server updates the TPPT in real time based on the feedback of the prediction results. In V2V communication, the vehicle offloads the real-time trajectory and downloads the overhead prediction results. The computational tasks and prediction results are transmitted to the predicted location of the edge server through the surrounding vehicles via V2V communication. Therefore, V2I and V2V communications are similar in the trajectory prediction process. In terms of task offloading, V2I communication transmits the prediction results and computation tasks to the predicted edge server through RSU, whereas V2V communication transmits the predicted results and computation tasks through wireless technology between vehicles.

We use the prediction time as the optimization objective of the prediction algorithm. Without incorporating the energy consumption, a computation task is defined as *T* [7], *T* = {*c*, *d*, *t_max_*}, where *c* is the number of computation resources required to complete the task, *d* is the size of the task, and *t_max_* denotes the maximum tolerable delay. The computation process of VEC usually considers 2 scenarios. If the computation task uses local computation resources, then it takes time *t*_1_ = *c*/*c_l_* to complete this task, where *c_l_* denotes the local computation resources. Since the local computing resource *c_l_* is usually small, it is difficult for time *t*_1_ to satisfy *t*_1_ < *t_max_*. The computation task can also be transmitted to the edge server *b*_0_. Therefore, the overall process requires time *t*_2_ = *c*/*c_s_* + *t_c_* + *t_y_*, where *c_s_* denotes the computational resources of the edge server, *t_c_* denotes the time of communication, and *t_y_* denotes the time of trajectory prediction. The vehicle trajectory prediction method based on the vehicle frequent pattern makes the total time *t*_2_ satisfy *t*_2_ < *t_max_* as much as possible by reducing *t_c_* and *t_y_*.

### 3.1. Definition of the T-Pattern Prediction Tree

The three data structures in the TPPT are the vehicle T-pattern tree, the vehicle frequent itemset and the vehicle frequent pattern. Among them, the vehicle T-pattern tree is composed of the prefixes of the vehicle T-pattern. The vehicle frequent itemset is composed of the suffixes of the vehicle T-pattern and their corresponding vehicle trajectory supports. The vehicle frequent pattern embodies the corresponding relationships of the vehicle T-pattern tree and the vehicle frequent itemset. The formal definition of the TPPT is shown below.

**Definition** **4.** *T-Pattern Prediction Tree. A T-pattern prediction tree is a ternary tuple containing a vehicle T-pattern tree, a vehicle frequent itemset and a vehicle frequent pattern*. *Given the vehicle trajectory datasets D, the T-pattern prediction tree can be represented as*

(9) $$TPPT=\left(\mathrm{Tree}\left(D\right),\;\mathrm{Item}\left(D\right),\;\mathrm{Frequent}\left(D\right)\right)$$

*where TPPT denotes the T-pattern prediction tree. Tree(D), Item(D) and Frequent(D) denote the vehicle T-pattern tree, the vehicle frequent itemset and the vehicle frequent pattern under the vehicle trajectory set D, respectively. In this paper, we provide a detailed explanation of the vehicle T-pattern tree, the vehicle frequent itemset and the vehicle frequent pattern*.

**Definition** **5.** *Vehicle T-Pattern Tree*. *A vehicle T-pattern tree is a tree structure consisting of a set of edge servers and a set of edges, formally defined as*

(10) $$TPT=\mathrm{Tree}\left(D\right)=\left(TB,E\right)$$

*where TB denotes the set of edge servers, i.e., N = {b_0_, b_1_, …, b_k_, …, b_n_}, b_k_ denotes the edge server in Equation (1); E is similar to the set of edges in Equation (4), i.e., E = {R_1_, R_2_, …, R_h_, …, R_m_}, R_h_ = (b_k−1_, b_k_, s_k_). In the vehicle T-pattern tree, the data structure is not a sequence. As a result, there is no direct relationship between the length of edge servers n and the length of edges m. In the vehicle T-pattern tree, node b_k_ is the child of node b_k−1_, and node b_k−1_ is the parent of node b_k_. If b_k_ does not have a parent node, b_k_ is the root node; if b_k_ does not have a child node, b_k_ is a leaf node.*

A branch of a vehicle T-pattern tree is a binary tuple. Specifically, given *TPT* = (*TB*, *E*), *TPT*′ = (*TB*′, *E*′), *N*′ ⊆ *N*, *E*′ ⊆ *E*, if ∀*b_k_* ϵ *N*′, *R_h_* ϵ *E*′ such that 1 ≤ |{*R_h_*|*b_k_* ϵ *R_h_*}| ≤ 2, *TPT*′ is a branch of the vehicle T-pattern tree *TPT*, denoted as *TR*.

In addition, the vehicle T-pattern tree needs to satisfy the following 3 conditions:

1. Each node has different children;
2. Each branch is a portion of the vehicle T-pattern;
3. All the branches starting with the root node and ending with the parent of the leaf node are the prefixes of the vehicle T-pattern.

**Definition** **6.** *Vehicle Frequent Itemset. The vehicle frequent itemset is the set consisting of the suffixes of the vehicle T-pattern and their corresponding vehicle trajectory support, i.e.*,

(11) $$IT=\mathrm{Item}\left(D\right)=\left\{set|set=\left(b,s\right)\right\}$$

*where set denotes the elements of the vehicle frequent itemset. b is the suffix of the vehicle T-pattern in Equation (6), denoting the key of set. s is the vehicle trajectory support in Equation (3), denoting the value of set*.

According to Definition 3, there exists a derivation relation which is also called the vehicle frequent pattern in Equation (7) between the prefix and suffix of a vehicle T-pattern. In the TPPT, we define the vehicle frequent pattern integrating the vehicle T-pattern tree and the vehicle frequent itemset as follows.

**Definition** **7.** *Vehicle Frequent Pattern*. *A vehicle frequent pattern is a set consisting of branches of a vehicle T-pattern tree and suffixes of a vehicle frequent itemset, which is formally defined as*

(12) $$FR=\mathrm{Frequent}\left(D\right)=\left\{U|U=(TR,b)\right\}$$

*where U is the vehicle frequent pattern between TR and b, TR is the branch of the vehicle T-pattern tree, and the edge server b is the key of the vehicle frequent itemset*.

To compare the advantages of the vehicle T-pattern prediction tree in Definition 4 and the vehicle T-pattern tree in Definition 5, taking the Capital Bikeshare datasets as an example, we construct the traditional T-pattern tree in [17,18] and the vehicle T-pattern tree which is a portion of the vehicle T-pattern prediction tree.

**Figure 3:**
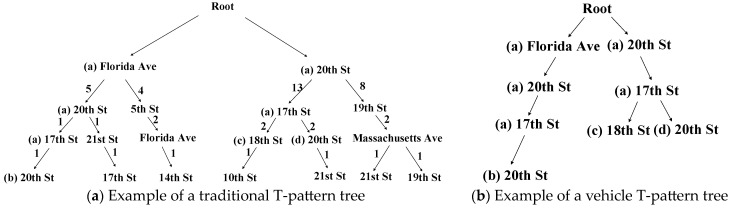
Example of a traditional T-pattern tree and a vehicle T-pattern tree.

Figure 3 shows examples of a traditional T-pattern tree and a vehicle T-pattern tree. The main difference between the traditional T-pattern tree in Figure 3a and the T-pattern prediction tree in Figure 3b is the storage content and the prediction process. In terms of storage content, the traditional T-pattern tree stores all of the historical trajectories, while the TPPT only stores the historical trajectories related to the current edge server. Therefore, the TPPT saves storage space and improves prediction efficiency. In terms of the prediction process, the traditional T-pattern tree traverses and searches for the node with the largest support of the vehicle trajectory, while the TPPT searches all of the leaf nodes through the vehicle frequent pattern and mines the leaf node with the largest support of the vehicle trajectory through the vehicle frequent itemset. Therefore, the TPPT optimizes the prediction process.

The TPPT consists of three parts: (1) Figure 3b shows a vehicle T-pattern tree. (2) Table 1 shows an example of a vehicle frequent itemset. (3) The branch of the vehicle T-pattern tree in Figure 3b and the key of the vehicle frequent itemset in Table 1 constitute the vehicle frequent pattern.

In the VEC scenario, we assume that the TPPT is stored in the edge server “17th St” which is the name of the edge server. Then, the TPPT maintained on the edge server is a data structure that consists of the vehicle T-pattern tree in Figure 3, the vehicle frequent itemset in Table 1, and the vehicle frequent pattern formed between them. Given the current vehicle trajectory formed by nodes (a) in Figure 3b and its corresponding edges, nodes (b), (c) and (d) in Figure 3b are potential following edge servers. The vehicle frequent itemset shown in Table 1 also consists of these potential following edge servers.

### 3.2. Construction of the T-Pattern Prediction Tree

In order to deploy the TPPT faster in VEC, this paper designed an algorithm for the construction of the TPPT. This algorithm is an initialization algorithm running on the edge server to optimize task offloading in VEC. At this initialization stage of V2I and V2V communications, the edge server downloads the historical data related to it from the cloud server and deploys the TPPT according to the construction algorithm in its memory. The Algorithm 1 is shown below.
**Algorithm 1:** Algorithm for construction of the T-pattern prediction tree.Input: vehicle trajectory datasets *D*, *D* = {*TB*_1_, *TB*_2_, …, *TB_j_*, …, *TB_z_*}.Output: T-pattern prediction tree *TPPT*.1. Init(*TPT*, *IT*, *FR*)2. for each *TB_j_* in *D*3.    for each *b_k_* in *TB_j_*4.      *TB_k_* = <*b*_0_, …, *b_k_*>5.      *TP_k_* = convert_TP(*TB_k_*)6.      if *TP_k_* in *TPT*:7.        *TP_i_* = *TPT*.find_branch(*TP_k_*)8.        *TP_i_*.support += 19.      if *TP_k_* not in *TPT*:10.        *TP_i_*_−1_ = *TPT*.find_branch(*TP_k_*_−1_)11.        *TP_i_* = *TP_i_*_−1_. Add_node(*b_k_*)12.        *TP_i_*.support = 113.    end for14. end for15. for *b_k_* in *TPT.Leaf_node*16.   *TB_k_* = <*b*_0_, …, *b_k_*>17.   *TP_k_* = Convert_TP(*TB_k_*)18.   *set_k_* = (*b_k_*, *TP_k_*.support)19.   IT.Add_set(*set_k_*)20.   FR.Add_relationship(*TP_k_*, *b_k_*)21. end for22. return *TPPT* = (*TPT*, *IT*, *FR*)

Among them, lines 2~14 construct the vehicle T-pattern tree. Lines 2~3 traverse each edge server *b_k_* of the vehicle trajectory *TB_j_*. Lines 4~5 construct the vehicle trajectory *TB_k_* and its corresponding vehicle T-pattern *TP_k_*, with *b*_0_ as the start and *b_k_* as the end. Lines 6~12 determine whether there is the same vehicle T-pattern as *TP_i_* in the vehicle T-pattern tree. If there is, the vehicle trajectory support corresponding to *TP_i_* is incremented; otherwise, *TP_i_* is constructed in the vehicle T-pattern tree and the vehicle trajectory support corresponding to *TP_i_* is set to 1.

Lines 15~22 construct the vehicle frequent itemset and the vehicle frequent pattern. Lines 15~17 traverse each leaf node *b_k_* of the vehicle T-pattern tree to construct the vehicle trajectory *TB_k_* and its corresponding vehicle T-pattern *TP_k_* starting from *b*_0_ and ending at *b_k_*. Lines 18~19 construct a set composed of the leaf node *b_k_* and the corresponding vehicle trajectory support. Then, the set is added into the vehicle frequent itemset. Line 20 constructs a binary tuple jointly constituted by the vehicle T-pattern *TP_k_* and the leaf node *b_k_*. Then, the binary tuple is added into the vehicle frequent patten.

For the construction process of the *TPPT*, we take the Capital Bikeshare datasets as an example, which is shown in Figure 3. In the example, after cleaning the data from the Capital Bikeshare datasets, the *TPPT* is constructed according to Algorithm 1. Specifically, the edge server “17th St” maintains this *TPPT*. The edge server “20th St” and “18th St” are the leaf nodes, which are the elements in the vehicle frequent itemset.

### 3.3. Prediction Based on the T-Pattern Prediction Tree

The TPPTs maintained by edge servers are designed to optimize predictive-mode task offloading in VEC. In this section, this paper proposes a prediction method based on the TPPT, in order to meet the real-time, accuracy and efficiency requirement of VEC. The prediction method is based on statistical theory. It needs to satisfy one assumption: assuming that a vehicle trajectory appears multiple times, then it can be assumed that this vehicle trajectory is related to the user’s driving habits. This assumption is applied in the TPPT as follows.

Both V2V and V2I communications require maintaining a T-pattern prediction tree. In V2I and V2V communications, a vehicle offloads the current trajectory to the edge server when it passes the edge server. The edge server matches the vehicle trajectory in the maintained vehicle T-pattern tree and finds the vehicle frequent itemset based on the matched vehicle trajectory and its vehicle frequent pattern. Then, the edge servers of the vehicle frequent itemset and their corresponding vehicle trajectory support are counted, and the edge server with the largest vehicle trajectory support is selected to accomplish the final prediction task.

The vehicle trajectory support in the vehicle frequent itemset indicates the number of occurrences of the trajectory. When predicting the trajectory, the same edge servers in the vehicle frequent itemset can be merged, and the corresponding vehicle trajectory support needs to be accumulated. In this paper, the accumulated result is called the support score, denoted as *score*. Its calculation formula is as follows:

(13) $$\mathrm{score}=\mathrm{predict}\left(b_{p}\right)=\sum_{set.\mathrm{key}=b_{p}}set.\mathrm{value}$$

where *set*.key is the former element of the *set* in the vehicle frequent itemset, i.e., the edge server *b_p_*. *set*.value is the latter element of the *set* in the vehicle frequent itemset, i.e., the vehicle trajectory support *s_p_*. The optimization scheme is to find the edge server that has the highest support score among the vehicle frequent itemset, which can also be represented as

(14) $$b_{p+1}=\underset{\mathrm{set}.\mathrm{key}}{\mathrm{argmax}}set.\mathrm{value}$$

where *b_p_*_+1_ is the prediction result, i.e., the edge server that the vehicle trajectory will pass through in the future. For this optimization scheme, this paper designed a prediction algorithm for the T-pattern prediction tree, also called the TPPT algorithm. The Algorithm 2 is shown below.
**Algorithm 2:** Prediction algorithm for the T-pattern prediction tree.Input: T-pattern prediction tree *TPPT*, current vehicle trajectory *TB_p_*.Output: Edge computing server *b_p_*_+1_.1. *TPT* = extract_TPT(*TPPT*)2. while *IT* = Ø do:3.    *TB* = match_TB(*TPT*, *TB_p_*)4.    *IT* = match_IT(*TB*, *FR*)5.    *TB_p_* = delete_first_node(*TB_p_*)6. end while7. merge(*IT*)8. Highest_score = 09. for each *set* in IT:10.    if *set*.value > Highest_score:11.      *b_p_*_+1_ = *set*.key12.      Highest_score = *set*.value13. end for14. return *b*_p+1_

Among them, lines 1~7 obtain the vehicle T-pattern tree and match the current vehicle trajectory in the vehicle T-pattern tree. Lines 3~4 find the vehicle frequent itemset related to the current vehicle trajectory through the vehicle frequent pattern. Then, that portion of the vehicle frequent itemset is merged according to the name or the serial number of the edge server. Lines 2~6 loop to match the current vehicle trajectory. If no vehicle frequent itemset is found, the first node of the vehicle trajectory is deleted. Lines 9~13 iterate through each *set* to find the *set* with the highest support score as the final prediction result.

In the example in Figure 3, the support scores corresponding to “18th St” and “20th St” are calculated and updated separately. The maximum value is taken as the highest support score. In Figure 3, “20th St” has the highest support score of 3. Finally, the edge server corresponding to the highest support score is output as the final prediction result. In Figure 3, “20th St” is the final prediction result.

### 3.4. Real-Time Updating of the T-Pattern Prediction Tree

The vehicle trajectories have a certain degree of effectiveness. To ensure the accuracy of the vehicle trajectory prediction, the T-pattern prediction tree maintained by edge servers needs to be updated in real time. For this reason, this paper designed a real-time updating algorithm for the T-pattern prediction tree. The application scenario of this method is as follows.

Given the prediction result *b_p_*_+1_ of the edge server *b_p_* and the time threshold *t,* if the current vehicle has passed the edge server *b_p_*_+1_ within the time threshold *t*, the prediction result is correct. Otherwise, the prediction result is incorrect. The edge server *b_p_*_+1_ generates feedback of the prediction result based on whether the prediction result is correct or not. The feedback is transmitted to the edge server *b_p_*. The edge server *b_p_* updates the maintained TPPT after obtaining the feedback. The Algorithm 3 is shown below.
**Algorithm 3:** Real-time updating algorithm for the T-pattern prediction tree.Input: original T-pattern prediction tree *TPFT_α_*, feedback of prediction result.Output: updated T-pattern prediction tree *TPFT_β_*.1. *TB* = match_TB(*TPT*, result.*TB*)2. *IT* = match_IT(*TB*, *FR*)3. if result.prediction = True:4.    *TB*.support += 15.    *IT*.set.value += 16. if result.prediction = False:7.    *TB*.support −= 18.    *IT.set*.value −= 19. *TPFT_β_* = update(*TPFT_α_*, *TB*, *IT*)10. return *TPFT_β_*

Among them, lines 1~3 obtain the vehicle T-pattern tree and the vehicle frequent itemset in the T-pattern prediction tree *TPFT_α_*. Lines 4~9 determine whether the prediction result is correct or not. If the prediction result is correct, the vehicle trajectory support corresponding to this vehicle trajectory is incremented. Otherwise, the vehicle trajectory support corresponding to this vehicle trajectory is decremented. Line 10 synchronizes the update results to the T-pattern prediction tree.

Taking the Capital Bikeshare datasets as an example, as shown in Figure 3, assume that the vehicle trajectory is <“20th St”, “17th St”> and the prediction result is “20th St”. If the vehicle trajectory subsequently passes through the edge server “20th St”, then the vehicle trajectory support corresponding to this vehicle trajectory in the TPPT is incremented. Otherwise, the vehicle trajectory support corresponding to this vehicle trajectory is decremented.

### 3.5. Task Offloading Strategies, Energy Consumption and Search Algorithm

In this section, we propose two task offloading strategies based on the proposed prediction method in Section 3.3. In addition, the incorrect prediction is considered in the strategies. After that, by analyzing the energy consumption, we also present a task offloading optimization algorithm, which minimizes the energy consumption with the time constraints.

Refs. [7,8] proposed the predictive-mode task offloading method via V2I and V2V, respectively. However, the trajectory was random. They did not integrate the trajectory prediction in the task offloading. In our task offloading strategies, we integrate the proposed prediction method. Before the task offloading, each edge server is initialized using Algorithm 1. The task offloading strategy in V2I communication is divided into 4 steps.

Step 1. When the vehicle arrives within the transmission range of the RSU, the computation tasks and the prediction tasks are offloaded to the edge server.

Step 2. The edge server predicts the location of the next edge server based on Algorithm 2. The computation tasks and/or their results are transmitted to the predicted edge server via V2I based on the search algorithm (which is introduced in the following part of this section).

Step 3. If the tasks are completed on two edge servers and the prediction result is correct, the results of the computation tasks are transmitted to the vehicle once it arrives. If the tasks are not completed on these two edge servers and the vehicle arrives within the transmission range of the last edge server, then go back to Step 2 and repeat. If the prediction result is incorrect and detected by Algorithm 3, the computation tasks and/or their results have to be transmitted to the known correct edge server via V2I communication.

Step 4. If the computation tasks and/or their results are transmitted to the known correct edge server, but the vehicle has been out of the transmission range, then go back to Step 2 and repeat.

The task offloading strategy in V2V communication is divided into 4 steps.

Step 1. When the vehicle arrives within the transmission range of the RSU, only the prediction tasks are offloaded to the edge server

Step 2. The edge server also predicts the location of the next edge server. Then, the prediction results are transmitted to the vehicle. After that, the computation tasks are transmitted to the predicted edge server via V2V communication based on the search algorithm.

Steps 3 and 4. The process is similar to that in V2I. The only difference is that the communication method is V2V.

For the energy consumption, we improve the definition in [7,8]. First, we consider the computation tasks is a set *C* = {*T*_1_, *T*_2_, …, *T_v_*, …, *T_w_*}. Each task *T_v_* = {*c_v_*, *d_v_*, *t_v_*} is a ternary tuple *T_v_* where *c_v_* is the number of computation resources required to complete the task *T_v_*, *d_v_* is the size of the task, and *t_v_* denotes the maximum tolerable delay. Then, for each edge server, *b* is a quintet defined as $b=\{f_{b},P_{b}^{c},P_{b}^{c}\}$, where $f_{b},P_{b}^{c},P_{b}^{c}$ are the computation rate, computation power and transmission power, respectively. Finally, let *f_r_* be the transmission rate.

According to the above definition, the computation time is

(15) $$t_{b}^{v}=\frac{c_{v}}{f_{b}}$$

and the transmission time is

(16) $$t_{r}^{v}=\frac{d_{v}}{f_{r}}$$

The computation energy consumption is

(17) $$E_{b}^{v}=\frac{c_{v}P_{b}^{c}}{f_{b}}$$

and the transmission energy consumption is

(18) $$E_{r}^{v}=\frac{d_{v}P_{r}^{c}}{f_{r}}$$

According to the above strategies, we do not know how many hops it will take to complete the computation tasks. Based on the proposed prediction algorithm, we can predict the neighboring edge server. Therefore, time is used as a constraint to locally optimize the computation tasks. As a result, we calculate the time consumption and energy consumption of neighboring edge servers. The time consumption is

(19) $$t_{all}=\sum_{v=1}^{w}x_{f}^{v}\left(t_{b_{1}}^{v}+t_{r}^{v}\right)+\left(1-x_{f}^{v}\right)t_{b_{2}}^{v}+t_{p}$$

where *t_p_* denotes the time consumption of the prediction, and the energy consumption is

(20) $$E_{all}=\sum_{v=1}^{w}x_{f}^{v}\left(E_{b_{1}}^{v}+E_{r}^{v}\right)+\left(1-x_{f}^{v}\right)E_{b_{2}}^{v}+t_{p}P_{b}^{c}$$

where $x_{f}^{v}$ is a binary variable indicating the status of task *v*. In the time and energy consumption, the tasks’ allocation and computation are only executed on two edge servers. Meanwhile, only the transmission of computation tasks is calculated. The size of the results is usually so small that they can be ignored in transmission.

The optimization problem based on the proposed prediction method for task offloading can be formulated as

(21) $$\min\;E_{all}\;s.t.0<t_{all}<\min(t_{1},\ldots ,t_{w})$$

where the constraints are such that there is no localized out-of-delay.

Finally, we propose a search algorithm to address the optimization problem according to Equation (21). The search algorithm is to minimize the energy consumption in Equation (20) with the time consumption in Equation (19). The Algorithm 4 is as follows.
**Algorithm 4:** Search algorithm for task offloading.Input: computation tasks set *C*, edge server *b*_1_, edge server *b*_2_.Output: minimum energy consumption *E_min_*.1. *E_min_* = +ꝏ2. for each *v* in |*C*|:3.    *t_all_* = calculate *t* in Equation (19) based on *v*, *b*_1,_
*b*_2_4.    *E_all_* = calculate *E* in Equation (20) based on *v*, *b*_1,_
*b*_2_5.    if *t_all_* < min(*t*_1_, …, *t_w_*):6.      if *E_all_* < *E_min_*:7.        *E_all_* = *E_min_*8. return *E_all_*

Among them, lines 2~7 traverse the computation tasks to obtain the minimum energy consumption with the constraint of time. Lines 3~4 calculate the time and energy consumption at each iteration. Line 5 takes the constraint into account. Lines 6~7 obtain the minimum energy consumption.

## 4. Results and Discussions

In order to verify the accuracy and efficiency of the method proposed in this paper, real-vehicle datasets and the bicycle check-in datasets publicly available on the Capital Bikeshare’s website [24] were selected as the experiment datasets. RNN [13], Transformer [14] and T-pattern algorithms [17,18,19,20,21,22] were chosen as the baseline methods.

Among them, RNN [13] and Transformer [14] are deep learning algorithms. Comparative experiments were conducted with such algorithms to analyze the applicability of deep learning in VEC. T-pattern algorithms [17,18,19,20,21,22] belong to class of algorithms based on the vehicle frequent pattern. Comparative experiments were conducted to analyze the accuracy and efficiency of the TPPT algorithm proposed in this paper.

### 4.1. Experimental Environment, Datasets and Parameters

This section describes the experimental environment, datasets and parameters. The environment was the Windows 10 operating system, an i5-8279U CPU, and the experimental codes were all written and run via python 3.9.

The experimental datasets used in the experiment were divided into two parts. The first part was the real-vehicle datasets. The fields of these datasets contained the trajectory serial number and the bayonet serial number. The second part was the bicycle check-in datasets publicly available on the Capital Bikeshare’s website [24]. The fields of these datasets contained the duration of use, start date, end date, start location, end location, vehicle number and membership type. The nodes in the datasets were not completely adjacent to each other. It could be used to simulate edge servers, as well as to predict the next position of a trajectory. The specific description of the datasets is shown in Table 2.

When processing the test datasets in the experiment, trajectories of length *n* were divided into trajectories of length *n* − 1 (>2) and trajectory of length 1 (a node) as the to-be-predicted trajectories and the true prediction results, respectively. Trajectories could not be divided with a trajectory length less than or equal to two. The datasets did not have any trajectory with a length greater than or equal to seven. Therefore, the data length threshold λ was set to three, four, five, and six in the experiment. A *k*-fold cross validation was designed during the experiment to solve the problem in two aspects. In terms of data volume, the *k*-fold cross-validation solved the problem of less data volume for the real data. In terms of the scenario of VEC, the trajectory data in the real scenario had randomness. The test datasets in the *k*-fold cross-validation were mutually exclusive each time, which was more in line with the application of this scenario.

Finally, the evaluation index of the experiment was given according to a classification problem. The accuracy of the algorithm in the experiment was analyzed based on the evaluation index. Meanwhile, the prediction efficiency of the algorithm in the experiment was analyzed according to the program running time.

Given a total of *z* samples in the experimental datasets, this paper calculated the accuracy evaluation index in the experiments, namely,

(22) $$Accuracy=\sum_{j=1}^{z}\frac{T_{j}}{T_{j}+F_{j}}$$

where *T_j_* denotes the number of samples predicted correctly, and *F_j_* denotes the number of samples predicted incorrectly in sample *j*. The experiment analyzed the accuracy by this formula and the efficiency by the running time of the program.

### 4.2. Comparisons and Analysis with Deep Learning Methods

Comparisons with deep learning methods used real-vehicle datasets. In the TPPT algorithm, the experimental results were taken as the highest and average values of accuracy and efficiency for different values of λ. The RNN and Transformer models used default hyperparameters. The experimental results are shown in Table 3.

In terms of accuracy, the TPPT algorithm reached the highest accuracy when λ was three, at which time it was better than the deep learning algorithms. However, the average accuracy of the TPPT algorithm was not as good as the deep learning algorithms. Typically, the length of the trajectory can be determined by users in VEC. Therefore, if users often offload trajectories with shorter lengths, the highest accuracy is more important in VEC. In order to further validate the applicability of deep learning methods, the experiment recorded the time of the training process and prediction process of each algorithm. The results are shown in Table 4.

The unit of time in Table 4 is the second. The TPPT algorithm proposed in this paper was much better than the deep learning algorithms in terms of training and prediction efficiency. In the training process, the training time of the algorithm proposed in this paper was about 78 times less than that of the deep learning algorithms. In the prediction process, the prediction time of the algorithm proposed in this paper was about 152 times less than that of the deep learning algorithms. In the scenario of VEC, the deep learning algorithms need to spend a lot of time to train the model, which cannot meet the real-time prediction requirement. Therefore, deep learning algorithms are not suitable for application in the scenario of VEC.

### 4.3. Comparisons and Analysis with the T-Pattern Algorithm

#### 4.3.1. Accuracy Comparisons and Analysis

In the accuracy analysis of the algorithm, relevant experiments were firstly conducted on the real-vehicle datasets. During the prediction process of the experiment, the number of correct and incorrect amounts was recorded when λ was three, four, five and six. The experimental results are shown in Table 5.

**Figure 4:**
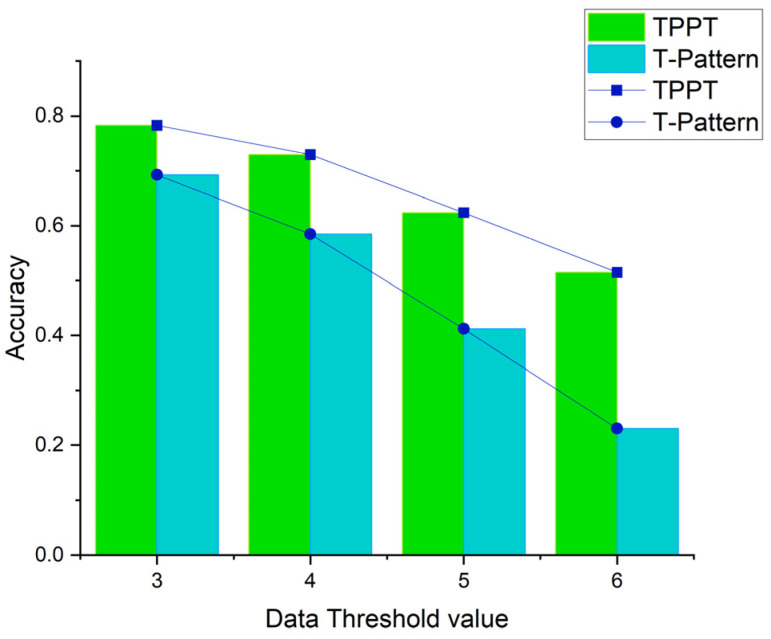
Schematic diagram of the prediction accuracy in the real-vehicle datasets.

According to the evaluation index, the accuracy of the three-fold cross-validation was calculated. The results are shown in Figure 4. Overall, in terms of accuracy, the prediction effect of the TPPT algorithm was better than that of the T-pattern algorithm. The accuracy was improved by more than 10%. When λ became large, compared with the T-pattern algorithm, the accuracy improvement of the TPPT algorithm became larger, but the accuracy decreased. After analyzing the incorrect data, it was found that this was because there were some trajectories that had not appeared in the historical trajectories. When λ became large, the number of such data increased, and the accuracy decreased. Meanwhile, when λ became larger, the T-pattern prediction tree had more geographic information, which led to the accuracy improvement becoming larger.

Then, experiments were carried out on the Capital Bikeshare datasets to again analyze the accuracy. In these datasets, 3 months of vehicle trajectories were selected as the training set and an adjacent month of vehicle trajectory was selected as the test set. The prediction task in datasets is to predict the edge servers that will be passed in the future given any trajectory whose nodes may not adjacent. The experimental results are shown in Table 6.

On the Capital Bikeshare datasets, the TPPT algorithm had more than twice the number of correct predictions compared with the T-pattern algorithm. It can be noticed that the number of correct predictions kept increasing over time. This was due to the increasing quantity of data in the training set. Both the T-pattern prediction tree and the T-pattern tree became larger and contained more information. Overall, there was a large amount of randomness in the latter month’s vehicle trajectory used for the prediction. The prediction result could be any one of thousands of nodes. The reason for the better prediction results of the TPPT algorithm is that the T-pattern prediction tree optimizes the statistical results. Specifically, the node with the highest probability of all the nodes is calculated in combination with the historical data.

#### 4.3.2. Efficiency Comparisons and Analysis

Experiments were carried out on the real-vehicle datasets to analyze the prediction efficiency of the algorithms proposed in this paper. During the experiments, the running time of different algorithms with different λ’s was recorded as different values. The results are shown in Table 7.

The experimental results showed that the TPPT algorithm had a substantial improvement in prediction efficiency, which was improved by more than 6.5 times relative to the T-Pattern prediction algorithm. This is because the vehicle T-pattern tree in the TPPT algorithm merges the repeated trajectories. Moreover, the T-pattern prediction tree prestores the prediction results, which reduces the time complexity and improves the prediction efficiency.

In addition, the threshold λ had a large impact on the algorithm’s prediction efficiency. This is because the threshold λ determines the size of the data volume and the size of the TPPT. In other words, at a lower threshold λ, the data volume is larger. The T-pattern prediction tree also has a larger size resulting in a longer running time. In the real environment, the TPPT only stores the trajectories related to the current edge server without traversing the full number of historical trajectories. Therefore, the algorithm proposed in this paper has a higher prediction efficiency in the real environment.

### 4.4. Performance Evaluation of Task Offloading

The above experiment demonstrates the performance of the prediction method. According to the proposed prediction method, we show illustrative results to demonstrate the performance of our proposed strategies and optimization method. Similar to [7,8], we compared with a local execution to analyze our time and energy consumption. We set similar parameters for the task offloading as in [8]. The detailed parameters are shown in Table 8.

In Table 8, the time consumption of the prediction is the average prediction time in Table 4. The time and energy consumption were calculated through Equations (15)–(20). During the experiments, we considered that the neighboring servers completed the corresponding computation tasks before transferring the computation tasks to the third server. The waiting time of the third server was ignored. Meanwhile, due to the unavailability of network dynamics, we distributed the computation tasks equally among the edge servers.

**Figure 5:**
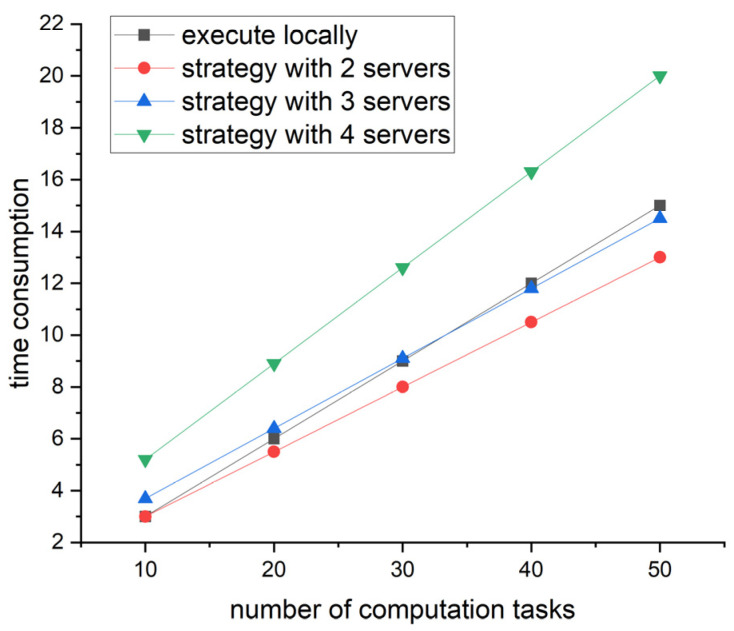
Line graph of time consumption and number of computation tasks.

In terms of time consumption, the number of edge servers was set to two, three, four and five in our proposed method. The time consumption was calculated when the number of computational tasks was 10, 20, 30, 40 and 50. The calculation results are shown in Figure 5.

Figure 5 shows the time consumption varies with the number of computation tasks under the four strategies. Overall, when the maximum tolerable delay is sufficient to complete the computation tasks, the proposed strategy with the least servers consumes the least amount of time. Namely, the strategy with two servers is superior to a local execution and the strategies with more servers. The experiments demonstrate that the strategy proposed in this paper consumes less time. Meanwhile, the experiments give an inspiration to us. When there are more tasks and not enough server resources and tasks have to be transmitted to multiple servers, the fewer servers used, the lower the time consumption. In detail, when the number of tasks is greater than 40, both the strategies with two and three servers outperform traditional local execution.

**Figure 6:**
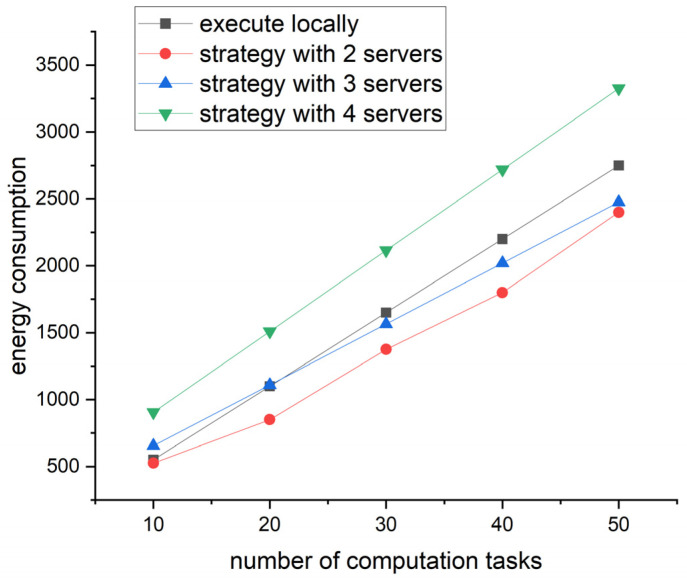
Line graph of energy consumption and number of computation tasks.

In terms of energy consumption, the setting was the same as that of time consumption. The calculation results are shown in Figure 6.

Figure 6 shows the energy consumption varies with the number of computation tasks under the four strategies. Overall, the strategy with two servers always has the lowest energy consumption, which proves that the strategy proposed in this paper is better than the traditional task offloading in terms of energy consumption. With regard to details, when the number of tasks is greater than 30, both the strategies with two and two servers outperform the traditional strategy. Meanwhile, the difference in energy consumption between the two strategies with two and three servers is small. Therefore, when optimizing the strategy, the strategy with the minimum number of servers should be selected. Similarly, in a dynamic network, if the number of servers is close, we need to select the best one based on real-time traffic.

## 5. Conclusions and Future Work

In order to accurately and efficiently predict vehicle trajectories in task offloading, a vehicle trajectory prediction framework based on the vehicle frequent pattern was proposed. At initialization, a T-pattern prediction tree is constructed based on historical data. In the prediction step, vehicle trajectories were predicted based on the T-pattern prediction tree. In real-time updating, the T-pattern prediction tree was updated based on the feedback of the prediction results.

The experiment was carried out on real-vehicle datasets and the Capital Bikeshare datasets. First, the experiment proved that deep learning algorithms were not suitable to the VEC scenario. Compared with the T-pattern algorithm, the accuracy of the proposed algorithm was improved by more than 10% and the efficiency was improved by more than 6.5 times. It could provide accurate and effective vehicle trajectory prediction results for task offloading in VEC.

In the future, we will conduct our research in three aspects.

- In terms of trajectory prediction, we will integrate more trajectory information to further improve the accuracy and efficiency, such as coordinate, timestamp and distance. Such information will affect the accuracy of trajectory predictions. In addition, such information is helpful in VEC, which affects the strategies of task offloading.
- In terms of task offloading in VEC, we will incorporate more of the energy consumption which is important in VEC. For the energy consumption, we may consider traffic dynamics and load balance in VEC. In addition, we will use the blockchain to manage the tasks.
- In terms of the styles of computing, we will try to use the TPPT in vehicular cloud computing and vehicular fog computing. Since different paradigms have different scopes of application, we will compare their application results.

## Figures and Tables

**Table 1:** Example of vehicle frequent itemset.

Key	Value
(b) 20th St	1
(d) 20th St	2
(c) 18th St	2
……	……

**Table 2:** Experimental datasets and their fields.

Data Sources	Field Name	Field Type
Real-vehicle datasets	Trajectory serial number	Unsigned integer
	Bayonet serial number	Unsigned integer
Capital Bikeshare	Duration of use	Unsigned integer
	Start date	String (computer science)
	End date	String (computer science)
	Start position	String/unsigned Integer
	End position	String/unsigned Integer
	Vehicle number	String (computer science)
	Membership type	String (computer science)

**Table 3:** Comparison of accuracy (%) of algorithms.

Algorithm	Maximum Accuracy	Average Accuracy
TPPT	78.3	66.3
RNN [13]	78.2	78.2
Transformer [14]	78.0	78.0

**Table 4:** Comparison of training and prediction efficiency of the algorithms.

Algorithm	Training Stage		Predicting Stage	
	Minimum Time	Average Time	Minimum Time	Average Time
TPPT	5.8904	43.6172	0.0045	0.5313
RNN	3355.9228	3355.9228	81.399	81.399
Transformer	3743.5721	3743.5721	698.6232	698.6232

**Table 5:** Prediction results on the real-vehicle datasets.

λ	Algorithm	Correct Amount	Incorrect Amount
3	TPPT	179,976	49,939
	T-Pattern	159,226	70,689
4	TPPT	21,006	7786
	T-Pattern	16,835	11,957
5	TPPT	3234	1949
	T-Pattern	2136	3047
6	TPPT	541	510
	T-Pattern	242	809

**Table 6:** Prediction results on the Capital Bikeshare datasets.

Training Set	TEST Set	Algorithm	Correct Number
Data from Jan to Mar	Data for Apr	TPPT	648
		T-Pattern	216
Data from May to Jul	Data for Aug	TPPT	837
		T-Pattern	230
Data from Sep to Nov	Data for Dec	TPPT	1084
		T-Pattern	478

**Table 7:** Time used for prediction in the real vehicle datasets.

λ	Algorithm	Running Time (s)
3	TPPT	1.9925
	T-Pattern	13.3361
4	TPPT	0.1048
	T-Pattern	1.7502
5	TPPT	0.0234
	T-Pattern	0.3809
6	TPPT	0.0045
	T-Pattern	0.0907

**Table 8:** Detailed parameters of task offloading.

Parameter	Description	Value
$c_{i}$	Computation load of task *i*	100
$f_{b}$	Computation rate of server *b*	500
$d_{i}$	Size of task *i*	20
$f_{r}$	Transmission rate	200
$P_{b}^{c}$	Computation power of server *b*	200
$P_{r}^{c}$	Transmission power of server *b*	150
$t_{p}$	Time consumption of prediction	0.5

## Data Availability

Please contact the corresponding author for available data support.
